# Nutritional composition and yield of forage grasses treated with vermicompost and urea

**DOI:** 10.1038/s41598-026-43372-4

**Published:** 2026-03-15

**Authors:** Enyew Mekcha, Bimrew Asmare, Netsanet Beyero, Shigdaf Mekuriaw

**Affiliations:** 1 Department of Animal Sciences, College of Agriculture and Environmental Sciences, Bahir Dar University, Bahir Dar, Ethiopia; 2 Department of Animal Science, College of Agriculture, Food and Climate Science, Injibara University, Injibara, Ethiopia; 3 International Livestock Research Institute (ILRI), Addis Ababa, Ethiopia

**Keywords:** Chemical composition, Crude protein yield, Dry matter yield, Profitability, Feed improvement, Ecology, Ecology, Environmental sciences, Plant sciences

## Abstract

Feed insecurity remains a major limiting factor to livestock production in Ethiopia. This study evaluated the effects of fertilizer treatments on the yield, nutritional composition, and economic returns of Napier (*Pennisetum purpureum*), Desho (*Pennisetum glaucifolium* Trin.), and Guinea (*Megathyrsus maximus*) grasses in northwestern Ethiopia. A factorial randomized complete block design with three replications was conducted at mid- and high-altitude sites. The treatments were: control, 100% vermicompost (VC), 70% VC + 30% urea, 30% VC + 70% urea, and 100% urea. Chemical composition parameters were analyzed, and crude protein yield per hectare (CPY t/ha) was quantified. The highest DMY (3.93 t ha⁻¹) and CPY (0.45 t ha⁻¹) of the grasses were recorded from 30% VC + 70% urea, followed by 70% VC + 30% urea. Sole VC produced moderate DMY (2.9 t ha⁻¹) but achieved the highest benefit–cost ratio (8.41). Mid-altitude conditions resulted in higher CP (9.6%) and CPY (0.37 t ha⁻¹) than high altitude. Napier grass recorded the highest CP (10.84%) and DMY (4.44 t ha⁻¹) among species. Integrated VC and urea maximized grasses yield, whereas sole VC represents a cost-efficient organic alternative for sustainable forage production and clean dairy value chains.

## Introduction

Ethiopia is home to the largest livestock population in Africa, with populations of over 70.3 million cattle, 42.9 million sheep, 52.5 million goats, and 57 million poultry^1^. The national livestock population in Ethiopia has steadily increased over recent years, and it is expected to keep rising, driven by the growing demand for animal-source products, including meat, milk, and eggs. It plays a vital role in supporting the livelihood of smallholder farmers, providing cash income, draft power, and essential inputs for crop production^1^. In addition, the sector contributes over 10% of the country’s total export earnings, mainly from the export of ruminant animals^2^. Despite its importance, livestock productivity in Ethiopia remains low, largely due to chronic shortages and seasonal fluctuation in the quantity and quality of available feed resources^3^.

Feed shortage is further aggravated by population growth, expansion of crop cultivation, and limited investment in forage production^3,4^. According to the report^1^, crop residues and natural pasture cover more than 78% of the national livestock feed resources, while improved forage covers only 0.2%, highlighting a substantial gap in the availability of quality feed. To address these challenges, several improved forage species have been introduced and promoted in Ethiopia over the past decades. These include grasses and legumes such as Rhodes grass (*Chloris gayana*), Oats–Vetch mixtures (*Avena sativa–Vicia* spp.), Alfalfa (*Medicago sativa*), Desho (*Pennisetum glaucifolium*), Napier (*Pennisetum purpureum*), Buffel grass (*Cenchrus ciliaris*), Sudan grass (*Sorghum sudanense*), Phalaris grass (*Phalaris aquatica*), and Guinea grass (*Panicum maximum*)^5^. Among these, cultivation of Desho, Guinea, and Napier with better agronomic practices has gained particular attention due to their high biomass yield, adaptability to a range of soil types and altitudinal zones, nutritional quality, and suitability for various feeding systems^3^. These improved forage grasses can reduce dependency on natural pasture and crop residue, increase herbage yield per unit area of production, and enhance livestock feed security^3^, as well as play a pivotal role in soil and water conservation^6^. Although cultivation of improved forage grasses plays a crucial role in enhancing the availability of livestock feed, soil fertility depletion continues to delay the quantity and quality of these improved forage grass species^7^.

Soil nutrient depletion affects the biomass yield and nutritional composition of forage grasses, which aggravates feed insecurity in Ethiopian farming systems. Therefore, fertilizer application enhances soil fertility as well as the yield and nutritional composition of forage grasses. In such cases, the application of inorganic fertilizers such as urea and diammonium phosphate (DAP) is commonly used to enhance forage yield and nutritional compositions^8^. However, their limited availability, high cost, potential for soil toxicity, and their predominant use on food crops limit their sustainable use for forage production by smallholder farmers. These limitations have increased interest in organic fertilizer sources, particularly VC, which is affordable, environmentally friendly, and capable of improving soil physicochemical properties^9^.

VC is produced through the biological decomposition of organic materials by earthworms (*Eisenia fetida* spp.) and is characterized by high nutrient availability, a low C: N ratio, and improved soil structure and nutrient retention^10^^,^^11^. Research results showed that the application of VC increases the supply of micronutrients to plants, in addition to mobilizing inaccessible nutrients into an accessible form. Moreover, VC reduces total Nitrogen loss, methane (CH_4_) and nitrous oxide (N_2_O) emissions, the cost of chemical fertilizer, and waste management emissions^12^. However, the sole application of VC may not meet the complete nutrient requirements of high-yielding forage grasses due to slow nutrient release patterns and a relatively imbalanced nutrient composition, particularly with respect to nitrogen^13^.

Given the high nutrient turnover rate in soil-plant systems, neither inorganic nor organic fertilizer alone can ensure sustainable forage production. Thus, the integrated application of VC and urea can synchronize nutrient release with plant demand, enhance soil fertility, and improve forage yield and nutritional composition^14^. Previous studies in Ethiopia have reported improvements in forage yield, morphological traits, and soil chemical properties under integrated VC and urea application^15,16^.

Available evidence on the combined effects of vermicompost and urea across multiple improved forage grass species and contrasting altitudinal zones in Ethiopia remains limited. Consequently, insufficient information exists on how sole and integrated applications of these fertilizers influence forage yield, nutritional composition, and economic returns under diverse agroecological conditions. This study contributes to the literature by evaluating these effects simultaneously across selected improved forage grass species in Ethiopia.

We hypothesized that the application of VC and urea, either alone or combined, would improve the yield, nutritional composition, and economic returns of forage grasses compared with unfertilized treatments. Therefore, this study was designed to evaluate the effects of sole and combined applications of VC and urea on yield, nutritional composition, and economic returns of Desho, Napier, and Guinea grasses grown at mid- and high-altitude locations in northwestern Ethiopia.

## Materials and methods

### Description of study areas

The experiments were conducted simultaneously during the main cropping season in two districts representing mid- and high-altitude locations in northwestern Ethiopia. The mid-altitude location was represented by North Mecha District (Ambomesk kebele), while the high-altitude location was represented by Banja District (Injibara University).

**Fig. 1:**
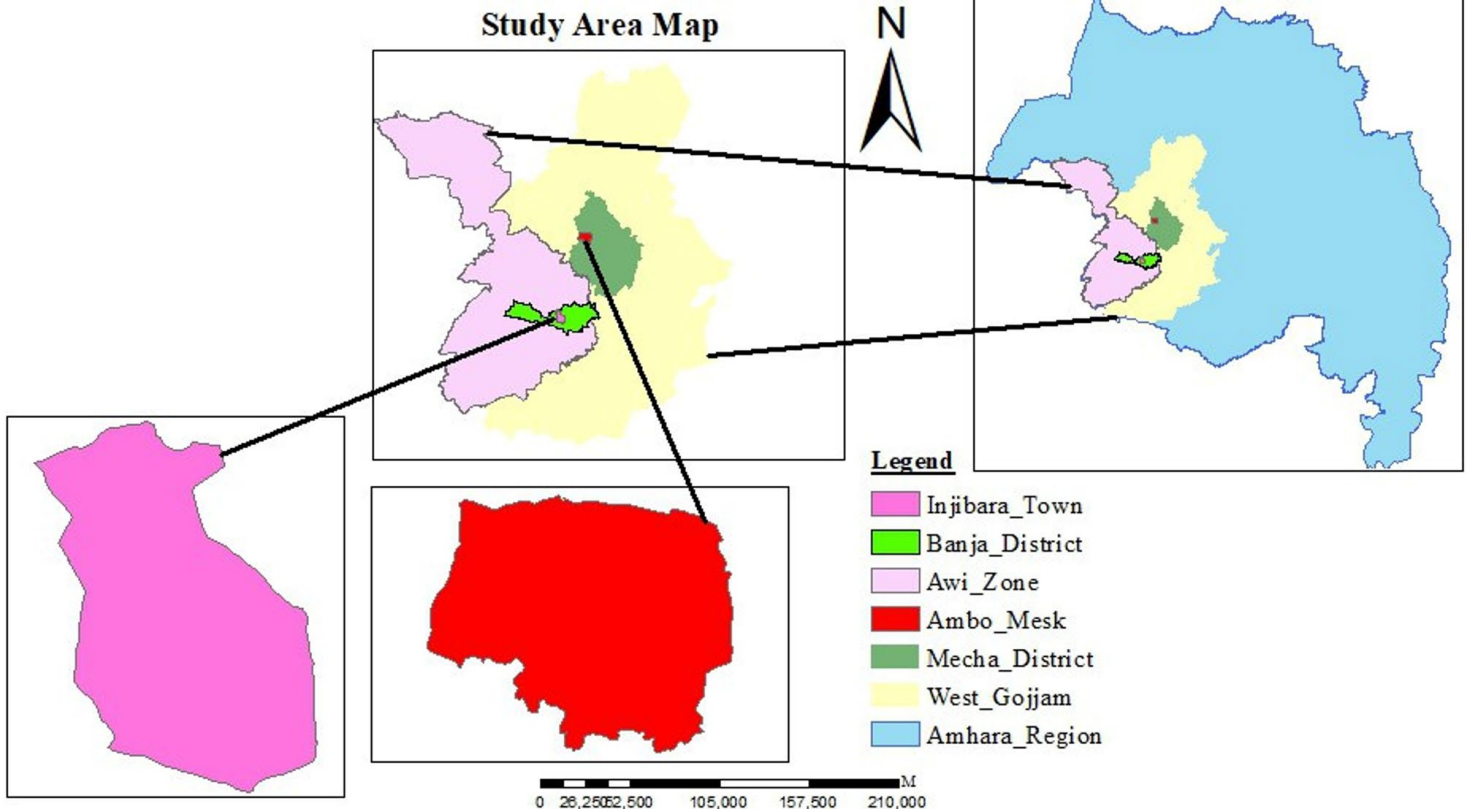
Location map of the study area. Generated using ArcGIS version 10.7.1 (Esri, Redlands, CA, USA; https://www.esri.com/en-us/arcgis/products/arcgis-desktop/overview).

North Mecha District is located approximately 35 km from Bahir Dar and 520 km northwest of Addis Ababa, the capital cities of the Amhara region and Ethiopia, respectively (Fig. 1). Geographically, the district lies between 11°05′N to 11°38′N latitude and 36°58′E to 37°22′E longitude, with an elevation ranging from 1800 to 2500 m above sea level (m.a.s.l.). The area receives a mean annual rainfall of approximately 3043.9 mm and experiences an average temperature of 23.5 °C. The dominant soil type is Nitisol, which is typically acidic with high levels of exchangeable acidity^11^. Inorganic fertilizers, particularly urea and Nitrogen-Phosphorus-Sulfur (NPS) blends, are widely used to enhance crop productivity. Furrow irrigation is a commonly practiced method in the district to supplement rainfall.

Banja District is found about 440 km northwest of Addis Ababa and 120 km southeast of Bahir Dar. It is geographically positioned at 10°56′17″N latitude and 36°52′16″E longitude, with an elevation of 2509 m.a.s.l. According to historical climatic data from the National Meteorological Service Agency (1984–2017), the district experiences a mean annual rainfall of approximately 1344 mm. The mean minimum and maximum temperatures of the area are 10.3 °C and 22.5 °C, respectively. The primary rainy season extends from June to September, followed by a secondary, less intense wet period continuing into November.

### Land preparation and experimental materials

The experimental land was initially cleared, harrowed, and plowed 20–30 days before plot layout and planting to remove unwanted weeds. Planting materials, such as Desho *(Pennisetum glaucifolium*, Kulumsa DZF-592), Napier (*Pennisetum purpureum acc. 15743)*, and Guinea (*Megathyrsus maximus*) grasses, were obtained from the Andassa Livestock Research Center. Only healthy and vigorous seedlings, free from physical damage and pest infestations, were selected for planting. Before transplanting, the leaves of the forage seedlings were trimmed to reduce transpiration and minimize transplanting shock. This step helped ensure better seedling establishment and survival under field conditions.

The vermicompost used in the study was prepared from cattle manure, green plant material, crop residues, and straw, following the guidelines of^17^. Wooden composting boxes of 1 m² were used, with each box lined with bedding materials (cow dung, straw, and leafy matter) and filled evenly. A mixture of cow dung and leafy matter was partially decomposed over five days before introducing the composting earthworms. Approximately 200 individuals of *Eisenia fetida* were introduced into each composting box, and the vermicomposting process was allowed to proceed for 60 days. The prepared VC had a carbon-to-nitrogen (C: N) ratio of 6.82:1, indicating a well-stabilized organic product suitable for soil application. Before the experiment began, the soil’s chemical properties were analyzed. At the mid altitude site, the soil had a pH of 5.31, organic carbon (OC) of 3.19 g/kg, total nitrogen (TN) of 0.19 g/kg, available phosphorus (AvP) of 2.74 ppm, organic matter (OM) of 2.41 g/kg, and cation exchange capacity (CEC) of 28.64 cmol/kg^15^. Corresponding values at the high-altitude pH, OC, TN, AvP, OM, and CEC were 5.04, 2.81 g/kg, 0.18 g/kg, 1.96 ppm, 2.12 g/kg, 24.69 cmol/kg, respectively. Urea fertilizer was purchased from the local market. Fertilizer was applied at the rate of 5 t/ha for VC and 100 kg/ha for urea. Specifically, VC was applied 35 days before planting, while urea was applied after forage establishment to avoid phytotoxicity.

### Treatments and experimental design

The experiment was conducted using a factorial randomized complete block design (RCBD), with replication at both mid and high altitude research sites. The factorial arrangement included three forage grass species and five fertilizer treatments, resulting 15 treatment combinations per site. Each experimental site comprised 45 plots measuring 3 × 3 m, with 1 m spacing between blocks and 0.5 m between plots. For planting, inter-row spacing of 0.5 m and intra-row spacing of 0.3 m was maintained, resulting in 60 plants per plot. The treatments used in the study are presented in Table 1.

**Table 1:** Treatment Combination.

Treatments	Level of application
T1	Control (no fertilizer)
T2	100% VC, 5 t/ha
T3	70% VC (3.5 t/ha) + 30% urea (30 kg/ha)
T4	30% VC (1.5 t/ha) + 70% urea (70 kg/ha)
T5	100% urea, 100 kg/ha

VC = vermicompost.

The amount of fertilizer applied to each plot was calculated based on the hectare application rate using the formula.

$$\text{Fertilizer per plot} = \frac{\text{Fertilizer rate/ha} \times \text{Plot area (m2)}}{10{,}000\ \mathrm{m2/ha}}$$

### Dry matter and crude protein yield measurement

Forage grasses were harvested at 105 days of growth, corresponding to the optimal stage for biomass and nutritional evaluation. Aboveground biomass was collected from a 1 m² area at the center of each plot, cutting at 8 cm above ground level. Immediately after harvesting, the fresh weight of the biomass was recorded in the field using a digital balance. A representative subsample of approximately 500 g of the grasses was air-dried under the shade and oven-dried at 65 °C for 72 h to determine the dry matter (DM) content following the procedure of^18^, and calculated as:

$$\text{DM (\%)} = \frac{\text{Oven dried weight (g)} \times 100}{\text{Fresh weight (g)}}$$

Dry matter yield (DMY, t/ha) was calculated as:

$$\normalsize \mathrm{DMY} = \text{Fresh weight (kg/m2)} \times \frac{\mathrm{DM}(\%)}{100} \times 10$$

and CPY (t/ha) of the grasses was calculated as:

$$\normalsize \text{CPY (t/ha)} = \mathrm{DMY} \times \frac{\mathrm{CP}(\%)}{100}$$

### Chemical analysis

The chemical analysis of forage grass was conducted at Jimma University College of Agriculture and Veterinary Medicine, specifically in the Animal Nutrition and Post-Harvest laboratories. The ash content was determined by igniting the oven-dried sample in a muffle furnace at 550°c for 4 h and calculated as ash (%)= weight of the residue (g)/dry sample weight  (g)​×100. 

The crude protein (CP) was analyzed according to the procedure of^18^. The Kjeldahl method determined nitrogen (N) content, and CP (%) was calculated as 6.25 x Nitrogen^18^. The methods of^19^ were used to determine neutral detergent fiber (NDF), acid detergent fiber (ADF), and acid detergent lignin (ADL).

### Cost-benefit analysis

Cost-benefit analysis followed^20^ to evaluate the economic feasibility of VC and urea applications. The analysis focused on evaluating variable costs and associated returns. The primary benefits were estimated on DM basis using the commercial market value of the DMY of the forage grasses, while all cost inputs were recorded at the start of the experiment. Fertilizer treatments (VC and urea) were considered the major sources of variable cost. The total variable cost (TVC) included expenses related to fertilizer acquisition and application. Total revenue (TR) was estimated by multiplying the DMY of each forage grass species (Desho, Napier, and Guinea) by the prevailing market prices (ETB 7,250 t/ha) prevailing during the study period. Net revenue (NR) was computed as the difference between total revenue and total variable cost.

### Statistical analysis

The collected data were subjected to the analysis of variance (ANOVA) using the general linear model (GLM) procedure in R software(R Studio version 4.3.2). Before ANOVA, model assumptions were evaluated. Normality of residuals was tested using the Shapiro–Wilk test and verified by visual inspection of Q–Q plots, while homogeneity of variances was assessed. When treatment effects were significant (*P* < 0.05), mean separation was performed using Tukey’s Honest Significant Difference (HSD) test. The statistical model for this design was:

$${\text{Yijkl }} = {\text{ }}\mu {\text{ }} + {\text{ Bi }} + {\text{ Fj }} + {\text{ Ak }} + {\text{ Al }} + {\text{ }}\left( {{\text{Fj }} \times {\text{ Ak}}} \right){\text{ }} + {\text{ }}\left( {{\text{Fj }} \times {\text{ Al}}} \right){\text{ }} + {\text{ }}\left( {{\text{Ak }} \times {\text{ Al}}} \right){\text{ }} + {\text{ eijkl}}$$

Where:

Yijkl = the response (dependent) variables.

µ = overall mean.

Bi = i^th^ block effect.

Fj = j^th^ factor effect (fertilizer treatment).

Ak = k^th^ factors effect (forage grasses).

Al = altitude effect.

Fj*Ak = interaction effect between fertilizer treatments and forage species.

Fj*Al = interaction effect between fertilizer treatment and altitude;

Ak*Al = interaction effect between forage grass species and altitude.

## Results

### Chemical composition and crude protein yield of forage grasses

The effects of fertilizer types, forage grass species, altitude, and their interaction on nutritional compositions and yield of forage grasses are presented in Table 2. The interaction between forage grass species and altitude significantly (*P* < 0.001) influenced ash, organic matter (OM), NDF, ADF, ADL, and CP concentration of forage grasses. However, the interaction between forage grass species and fertilizer types, forage species and altitude, and fertilizer type with altitude did not significantly (*P* > 0.05) affect the DM content of the grass. CPY was significantly (*P* < 0.001) affected by the interactions of forage grass species with altitude, forage species with fertilizer treatment, and fertilizer treatment with altitude.

Fertilizer application, whether combined or sole, significantly improved the nutritional composition of forage grasses. The highest CP content (11.07% and 11.04%) was observed under the combined application (T3 and T4), with no significant difference between them, while the control plots exhibited the lowest CP (7.81%). Fiber fractions (NDF, ADF, and ADL) contents of forage grasses were significantly affected by the application of fertilizer treatments. The lowest fiber fractions of the grasses were observed under the combined application compared to the sole application. CPY followed a similar trend, with the highest values observed under combined applications (3.93–3.86 t/ha) and the lowest in control plots (0.21 t/ha).

Altitude significantly influenced chemical composition and yield. Forage grasses grown at high-altitude sites had higher ash content (13.19%) compared to mid-altitude sites (11.95%) (*P* < 0.001). Conversely, grasses at mid-altitude had higher OM, NDF, ADF, ADL, CP, DMY, and CPY. CP content was 6.22% higher at mid-altitude, and CPY was 27.59% greater than at high-altitude.

Napier grass had significantly (*P* < 0.001) higher CP content (10.84%) compared to Guinea grass (9.05%) and Desho grass (8.15%). Guinea grass exhibited higher DM (34.7%), NDF (67.85%), ADF (40.44%), and ADL (10.09%) compared to Napier and Desho grasses, whereas Napier grass showed the highest OM (88.22%). In terms of yield, Napier grass had the highest CPY (0.49 t/ha), while Desho grass recorded the lowest (0.19 t/ha CPY).

**Table 2:** Chemical composition, dry matter yield, and crude protein yield of forage grasses as influenced by forage species, fertilizer type, and altitude.

Variables	DM (%)	Ash %DM	OM %DM	NDF %DM	ADF %DM	ADL %DM	CP %DM	DMY t/ha	CPY t/ha
***Altitudes***									
Mid	33.2^a^	11.95^b^	88.05^a^	65.44^a^	37.11^a^	9.44^a^	9.64^a^	3.59^a^	0.37^a^
High	32.01^b^	13.19^a^	86.81^b^	64.29^b^	36.03^b^	9.07^b^	9.04^b^	3.06^b^	0.29^b^
Sig	***	***	***	***	***	***	***	***	***
***Fertilizer treatments***									
T1	32.86	13.59^a^	86.41^c^	67.36^a^	38.62^a^	10.11^a^	7.81^c^	2.65^d^	0.21^c^
T2	32.89	12.61^b^	87.39^b^	66.50^b^	37.68^b^	10.01^b^	8.40^b^	2.90^c^	0.25^bc^
T3	32.03	10.98c	89.02^a^	62.68^c^	35.34^c^	8.33^c^	11.04^a^	3.93^a^	0.45^a^
T4	32.48	12.04^b^	87.96^b^	62.43^c^	35.32^c^	8.31^c^	11.07^a^	3.86^a^	0.44^a^
T5	32.77	13.63^a^	86.37^c^	65.36^b^	37.39^b^	9.51^b^	8.41^b^	3.29^b^	0.28^b^
Sig	ns	***	***	***	***	***	***	***	***
***Forage grass***									
Desho	31.09^c^	12.66a	87.34^b^	61.61^c^	34.71^c^	9.67^b^	8.15^c^	2.24^c^	0.19^c^
Napier	32.02^b^	11.79^b^	88.22^a^	65.13^b^	35.47^b^	8.01^c^	10.84^a^	4.44^a^	0.49^a^
Guinea	34.70^a^	13.27^a^	86.73^b^	67.85^a^	40.44^a^	10.09^a^	9.05^b^	3.29^b^	0.31^b^
Sig	***	***	***	***	***	***	***	***	***
**Grand mean**	32.61	12.57	87.43	64.86	36.87	9.25	9.34	3.33	0.33
CV (%)	3.15	4.19	0.6	1.27	1.26	3.35	3.62	2.81	5.68
***Interaction***									
Fg*Ft	ns	***	***	***	***	***	***	***	***
Fg*al	ns	ns	ns	ns	ns	ns	*	***	***
Ft * al	ns	ns	ns	ns	ns	ns	*	***	***
Fg*Ft*al	ns	ns	ns	ns	ns	ns	ns	ns	***

*=significant at 0.05, **=significant at 0.01, ***=significant at 0. 001, means within a row followed by the same letters are not significantly different, ns = non-significant, DM = Dry matter, OM = Organic matter, NDF = neutral detergent fiber, ADF = Acid detergent fiber, ADL = Acid detergent lignin, CP = Crude protein, DMY = Dry matter yield, CPY = Crude protein yield, CV = coefficient of variation, Fg = Forage grass, al = altitude, Ft = Fertilizer. T1 = Control, T2 = 100% VC, T3 = 70% VC + 30% urea, T4 = 30% VC + 70% urea, T5 = 100% urea.

### Interaction effect of forage grass species with fertilizer treatments

Table 3 presents the interaction effect between forage species and fertilizer treatments. The interaction between forage grass species and fertilizer treatments significantly affected the nutritional composition and yield of forage grasses. Napier grass responded most strongly to combined applications of VC and urea (T3 and T4), achieving the highest CP content (12.90–13.02%), DMY (4.94–5.10 t/ha), and CPY (0.64–0.67 t/ha). Guinea grass also showed improved CPY under combined applications, whereas Desho grass exhibited comparatively lower responses in CP and CPY across all fertilizer treatments.

**Table 3:** Interaction effects of forage grass species and fertilizer treatments on chemical composition, DMY, and CPY.

Forage grass	Treatments	Parameters								
		DM (%)	Ash %DM	OM %DM	NDF %DM	ADF %DM	ADL %DM	CP %DM	DMY t/ha	CPY t/ha
Napier	T1	32.58^bcde^	12.41^cd^	87.59^bc^	67.34^bc^	36.73^de^	9.18^de^	8.68^de^	3.78^cd^	0.328^cd^
	T2	32.37^cde^	12.34^cd^	87.66^bc^	66.14^cd^	36.20^ef^	9.11^e^	9.89^c^	3.99^c^	0.39^bc^
	T3	31.15^e^	11.05^d^	88.95^b^	63.68^ef^	34.55^fg^	6.88^g^	13.02^a^	5.10^a^	0.67^a^
	T4	31.73^de^	11.20^d^	88.81^b^	63.16^fg^	34.40^fg^	6.74^g^	12.90^a^	4.94^ab^	0.64^a^
	T5	32.30^cde^	11.89^cd^	88.11^bc^	65.34^cde^	35.46^ef^	8.12^f^	9.68^c^	4.40^bc^	0.43^bc^
Desho	T1	31.08^e^	14.24^ab^	85.76^de^	65.00^def^	36.82^cde^	10.09^bc^	7.32^f^	1.83^h^	0.13^f^
	T2	31.10^e^	11.91^cd^	88.09^bc^	64.02^ef^	35.44^ef^	10.18^bc^	7.47^f^	2.04^gh^	0.15^ef^
	T3	32.01^cde^	9.33^e^	90.67^a^	58.73^h^	32.75^g^	9.11^e^	9.20^cd^	2.54^efg^	0.23^de^
	T4	30.25^e^	12.36^cd^	87.64^bc^	58.96^h^	32.71^g^	9.07^e^	9.22^cd^	2.50^fg^	0.23^def^
	T5	31.03^e^	15.48^a^	84.52^e^	61.34^g^	35.81^ef^	9.91^cd^	7.55^f^	2.30^fgh^	0.17^ef^
Guinea	T1	34.92^ab^	14.13^ab^	85.87^de^	69.75^a^	42.30^a^	11.06^a^	7.44^f^	2.34^fgh^	0.17^ef^
	T2	35.19^a^	13.58^bc^	86.42^cd^	69.33^ab^	41.39^a^	10.75^ab^	7.81^ef^	2.69^ef^	0.21^ef^
	T3	34.29^abc^	12.57^bcd^	87.43^bcd^	65.63^cde^	38.73^cd^	8.99^e^	10.91^b^	4.15^c^	0.47^b^
	T4	34.13^abcd^	12.56^bcd^	87.44^bcd^	65.16^def^	38.87^bc^	9.13^e^	11.10^b^	4.13^c^	0.46^b^
	T5	34.98^ab^	13.51^bc^	86.49^cd^	69.39^ab^	40.89^ab^	10.50^abc^	7.99^ef^	3.16^de^	0.25^de^

Means within a row followed by the same letters are not significantly different, DM = Dry matter, OM = Organic matter, NDF = neutral detergent fiber, ADF = Acid detergent fiber, ADL = Acid detergent lignin, CP = Crude protein, DMY = Dry matter yield, CPY = Crude protein yield, CV = coefficient of variation, Fg = Forage grass, al = altitude, Ft = Fertilizer. T1 = Control, T2 = 100% VC, T3 = 70% VC + 30% urea, T4 = 30% VC + 70% urea, T5 = 100% urea.

### Correlation analysis of nutritional parameters and yield of forage grasses

The Pearson correlation analysis among the chemical composition and CP yield of forage grasses is presented in Table 4. As presented in the table, the DM content exhibited a significant positive correlation with ash, NDF, ADF, and the CPY. On the other hand, the DM content was negatively correlated with OM and CP content of forage grass. In the study, the ash content of the grass showed a perfect inverse relationship with OM (*r* = − 1.00). Moreover, the ash content of the grass was negatively correlated with CP and CPY. On the other hand, the ash content of the grasses was positively correlated with structural fiber fractions (NDF, ADF, and ADL). The OM content of the grasses showed a negative correlation with NDF, ADF, and ADL, while it was positively correlated with CP and CPY. Neutral detergent fiber content of the grasses was positively correlated with ADF and ADL contents, while it was negatively correlated with CP and CPY. The CP content of the grasses was positively and significantly correlated with the CPY.

**Table 4:** Correlation coefficients among nutritional composition and yield of forage grasses.

	DM	Ash	OM	NDF	ADF	ADL	CP	DMY	CPY
DM	1.0000								
Ash	0.0680	1.0000							
OM	− 0.0680	− 1.0000	1.0000						
NDF	0.6630	0.2981	− 0.2981	1.0000					
ADF	0.7719	0.3869	− 0.3869	0.8590	1.0000				
ADL	0.3966	0.4841	− 0.4841	0.4180	0.6253	1.0000			
CP	− 0.0631	− 0.5650	0.5650	− 0.2000	− 0.2876	− 0.8227	1.0000		
DMY	0.1354	− 0.4758	0.4758	0.1173	− 0.0613	− 0.7236	0.8782	1.0000	
CPY	0.0315	− 0.5092	0.5092	− 0.0146	− 0.1599	− 0.7909	0.9612	0.9660	1.000

DM = Dry matter, OM = Organic matter, NDF = Neutral detergent fiber, ADF = Acid detergent fiber, ADL = Acid detergent lignin, CP = Crude protein, DMY = Dry matter yield, CPY = Crude protein yield

### Estimated returns from the sale of forage grasses

A cost-benefit analysis was conducted to assess the economic viability of forage grass production under varying fertilization treatments and altitudinal zones, using revenue from forage biomass sales as the principal income source (Table 5). The results of the current study indicated that forage grasses cultivated at the mid-altitude location yielded NR compared to those grasses cultivated at the high-altitude location. Among the forage species evaluated, Napier grass generated the greatest NR, outperforming Guinea grass and Desho grass, which produced intermediate and lowest returns, respectively. Specifically, Napier grass cultivation resulted in 33.1% and 63.2% greater NR compared to Guinea grass and Desho grass, respectively. Regarding fertilizer treatments, the combined application of T3 yielded the highest NR over all treatments, followed by T4 and T5. Notably, the lowest NR was recorded under the sole application of urea, which was even lower than the control treatment.

**Table 5:** Partial budget analysis of forage grass as influenced by altitude, grass species, and fertilizer treatments.

Descriptions	DMY (t/ha)	Cost of Urea (ETB/ha)	Cost of VC (ETB t/ha)	TVC (ETB t/ha)	TR (ETB t/ha)		NR (ETB t/ha)	BCR
**Altitude**								
Mid	3.59	8, 000	2, 500	10, 500	26, 027.5	15, 527.5		2.48
High	3.06	8, 000	2, 500	10, 500	22, 185	11, 685		2.11
**Grass species**								
Desho	2.24	5, 333	1, 667	7, 000	16, 240	9, 240		2.32
Napier	4.44	5, 333	1, 667	7, 000	32, 190	25, 190		4.6
Guinea	3.29	5, 333	1, 667	7, 000	23, 852.5	16, 852.5		3.41
**Treatments**								
T1	2.65	0	0	0	19, 212.5	19, 212.5		-
T2	2.9	0	2, 500	2, 500	21, 025	18, 525		8.41
T3	3.93	2, 400	1, 750	4, 150	28, 492.5	24, 342.5		6.87
T4	3.86	5, 600	750	6, 350	27, 985	21, 635		4.41
T5	3.29	8, 000	0	8, 000	23, 852.5	15, 852.5		2.98

DMY = dry matter yield, ETB = Ethiopian birr, U = urea, VC = Vermicompost, TVC = Total variable cost, TR = Total return, NR = Net return, BCR = Benefit cost ratio, T1 = Control, T2 = 100% VC, T3 = 70% VC + 30% urea, T4 = 30% VC + 70% urea, T5 = 100% urea.

## Discussions

The present study initially hypothesized that either the sole or the combined application of VC and urea would improve the yield, nutritional composition, and economic returns of forage grasses. The result confirms that fertilizer application, particularly combined applications of VC and urea, significantly enhanced the nutritional composition and CPY of the grasses. The superior performance in the combined fertilizer application is likely due to their complementary nutrient-release patterns^21^. Urea provides readily available nitrogen that supports rapid vegetative growth, while VC supplies a sustained release of nitrogen supporting continuous protein synthesis during the later growth stages of the grass^22^. The absence of significant differences between T3 and T4 further indicates that moderate integration of VC and urea is sufficient to maximize CP accumulation, which has practical implications for reducing fertilizer costs without compromising forage quality. Similar improvements in CP content under integrated fertilizer application have been reported previously^23,24^. In contrast, the relatively lower CP content under sole fertilizer applications may reflect the absence of a synergistic nutrient release pattern.

Moreover, the CPY of the grasses was also higher in the combined fertilizer application, likely due to the combined effect of increased CP concentration and enhanced DMY in combined urea and VC application. Thus, integrated fertilization improved not only grass nutritional composition but also protein output per unit area, a critical indicator for livestock feeding systems. Previous studies have similarly reported that the combined application of VC and urea enhances the CPY of *Panicum coloratum* grass compared to individual application^16^.

Besides the CP concentration and yield, integrated fertilization also influenced fiber composition. The reduced NDF, ADF, and ADL contents under combined VC and urea application show a higher proportion of leaf relative to stem biomass. Field observations during the experiment indicated more vigorous vegetative growth and increased leaf density in integrated fertilizer plots, which may have shown stem lignification and reduced cell wall components. Such changes are nutritionally desirable, as lower fiber concentrations are generally associated with improved intake potential and digestibility^25^. Similar response has also been reported by^16,26^.

Altitude also significantly influenced the nutritional composition and the yield of forage grasses. The superior performance of forage grasses at the mid-altitude location can be explained by differences in climatic conditions, land characteristics, and soil physicochemical properties across altitudinal gradients. Significantly higher CP concentration in the altitudes is attributed to moderate temperature and favorable soil conditions at mid-altitudes, which enhanced nitrogen uptake and biomass production. In contrast, cooler conditions at higher altitudes may have reduced soil microbial activity and nutrient mineralization, thereby limiting nitrogen availability to plants, resulting in reduced nitrogen synthesis in^27^. Comparable altitude-related trends in forage CP content have been reported by^28,29^, although contrasting findings in other studies^30^ was observed.

The fiber fractions of forage grasses were also influenced by altitude variation. Higher NDF, ADF, and ADL contents observed in mid-altitude grasses may reflect faster growth rates under warmer conditions, which can accelerate cell wall thickening and lignification^27^. Conversely, cooler high-altitude conditions may slow lignification relative to stem elongation, resulting in comparatively lower fiber concentrations and potentially higher digestibility^31^.

Variation in the chemical composition among forage grass species was primarily attributed to inherent genetic differences that regulate nutrient uptake efficiency, adaptability to environmental conditions, interactions with soil microbiota, and morphological traits such as tillering capacity and leaf elongation^30^. These species-specific characteristics influence biomass partitioning between leaves and stems, thereby determining crude protein concentration and fiber accumulation. The observed difference in CP and CPY among grass species is consistent with earlier findings^29,32,33^. The difference in CPY among grass species is also likely due to variation in the CP and DMY between grass species. Relatively lower CPY in Guinea and Desho grasses is likely due to the lowest CP and DMY of the grasses compared to Napier grass.

The interaction between forage grass species and fertilizer in the present study indicated that the forage species responded differently to fertilizer treatments. Napier grass exhibited the strongest response to integrated VC and urea application, recording the highest CP concentration and CPY, showing its high nutrient uptake efficiency and vigorous vegetative growth under improved soil fertility conditions. In contrast, Desho grass showed a relatively modest response to fertilizer integration, particularly in terms of CPY, likely due to inherently lower biomass production potential and CP content. Guinea grass on the other hand has an intermediate response for CP and CPY, although the response was lower than that observed in Napier grass. These differential responses highlight the importance of matching fertilizer strategies with forage species characteristics to forage grass yield and quality under smallholder production systems.

A significant and positive correlation between DM with ash, fiber fractions (NDF, ADF, and ADL), DMY, and CPY showed that as grasses accumulate higher structural biomass, overall productivity also increases. In contrast, DM content showed significant negative correlations with OM and CP, reflecting the trade-off between biomass accumulation and forage quality, where increased structural development is associated with reduced nutritional value. The significant negative association between ash and OM, CP, DMY, and CPY might be explained by the inverse relationship between ash and organic components^19^. Furthermore, higher ash content was associated with less tillering, resulting in lower biomass accumulation. On the other hand, the OM content of the grasses was positively correlated with CP, DMY, and CPY. Forage with higher OM generally contain greater concentration of protein and digestible carbohydrates, enhancing DM accumulation and CPY. This relationship helps explain the superior CPY observed under integrated VC and urea application.

In contrast, OM content was negatively correlated with fiber fractions, indicating that increased accumulation of structural carbohydrates displaces nutrient-rich organic components. The strong positive correlations among NDF, ADF, and ADL confirm coordinated cell wall development. The negative associations between fiber fractions and CP and CPY demonstrate that increased lignification limits forage nutritive value and protein yield, supporting the observed benefit of combined fertilizer application.

The economic analysis of forage grass production revealed clear variations in profitability across altitudinal zones, forage species, and fertilizer treatments. Mid-altitude locations consistently produced higher profitability, likely due to a combination of favorable climatic conditions, moderate temperatures, adequate moisture, and higher nutrient-use efficiency. These conditions enhanced dry matter accumulation and optimized the response of forage grasses to fertilizer applications, translating into increased TR and NR per hectare.

Among the forage species, Napier grass consistently outperformed Desho and Guinea grasses in terms of TR, NR, and BCR. This superior performance can be attributed to its higher biomass yield and favorable leaf-to-stem ratio, which together improve both forage quantity and quality. Desho grass exhibited lower profitability despite similar input costs, reflecting its comparatively lower DMY and protein accumulation. These results emphasize that grass species selection is critical for maximizing economic returns in forage-based livestock systems.

Fertilizer application also has an effect on the economic returns of forage grass. The sole VC application resulted in the highest BCR, due to its low input costs and efficient conversion of inputs into economic returns. However, integrating VC with urea produced the highest absolute NR, highlighting the synergistic benefits of combining organic and inorganic fertilizers. The enhanced profitability under the combined application is explained by improved nutrient availability throughout the growth period, which supports higher biomass production, protein yield, and forage quality. Conversely, sole urea application substantially increased production costs without proportionate yield gains, demonstrating reduced economic efficiency and diminishing marginal returns. A similar trend was observed by^24^.

These findings demonstrate that integrated VC and urea application is cost effective strategy that maximizes both forage productivity and profitability, while emphasizing the need to consider species-specific and altitudinal responses when planning forage production interventions.

## Conclusion

This study demonstrates that VC and urea significantly improved DMY, CP content, CPY, and economic returns of Napier, Desho, and Guinea grasses across mid- and high-altitude environments in northwestern Ethiopia. Integrated application of VC and urea produced higher DMY and CPY, confirming its suitability for productivity-oriented forage systems, with Napier grass performing best at mid-altitude. However, sole VC (100%) generated the highest BCR, indicating that organic fertilization is the most economically viable option for smallholder farmers. Beyond its economic advantage, VC-based forage production reduces dependence on synthetic fertilizers and provides residue-free feed resources that support animal health and clean milk production, thereby contributing to human health and environmentally sustainable dairy development. Therefore, promotion of on-farm VC production should be prioritized through extension and development programs. Further multi-location and multi-season studies are recommended to validate these findings and assess long-term impacts on soil health, forage productivity, and livestock performance.

## Data Availability

The raw data involved in the analysis during the current study are available from the corresponding author upon reasonable request.
